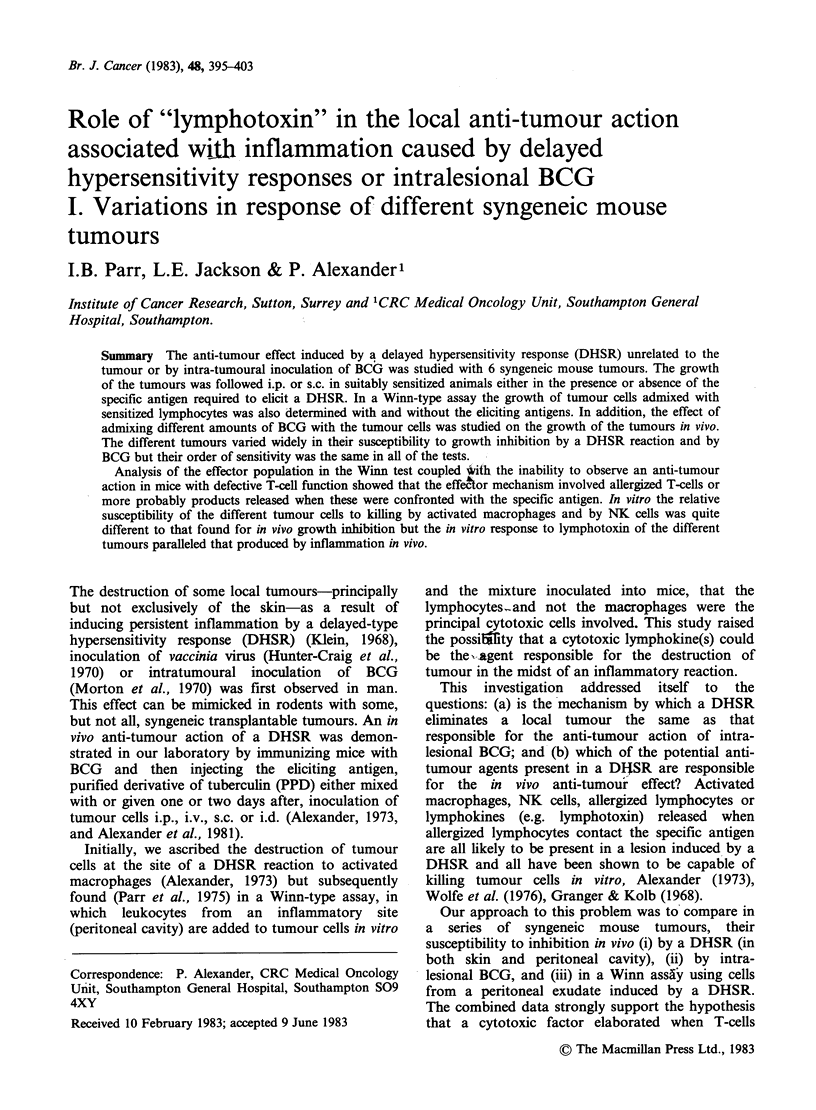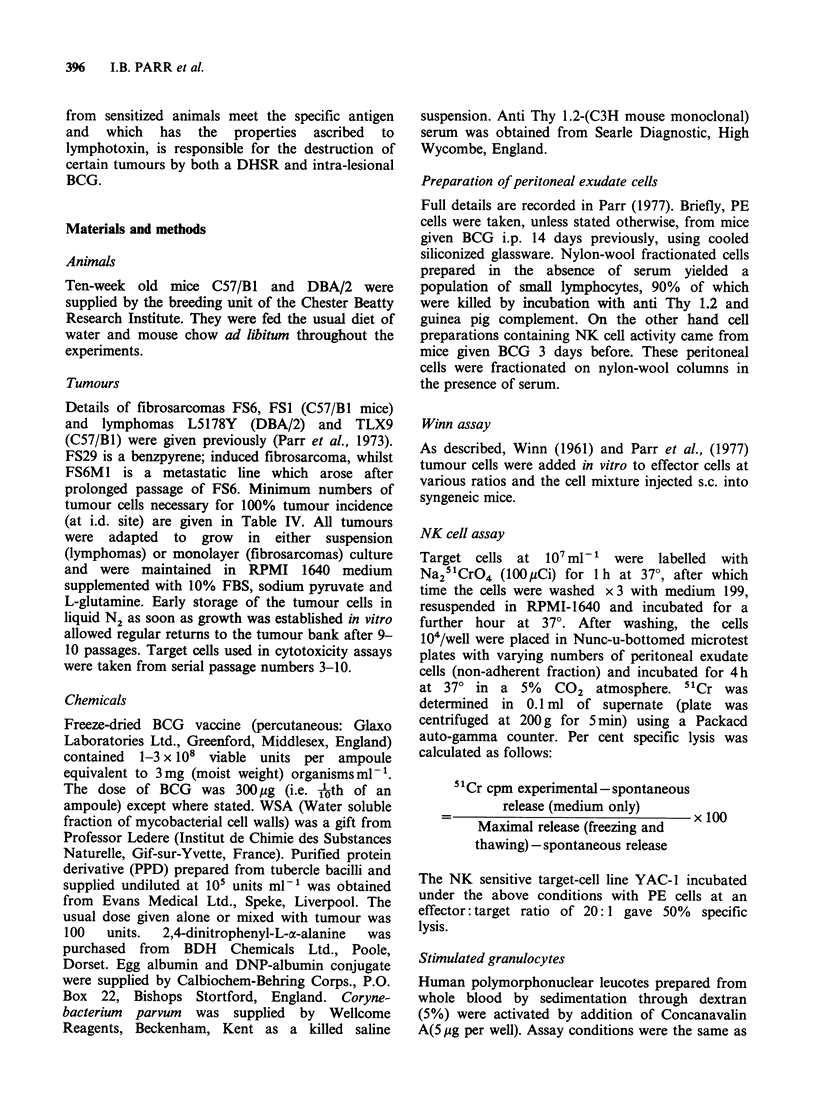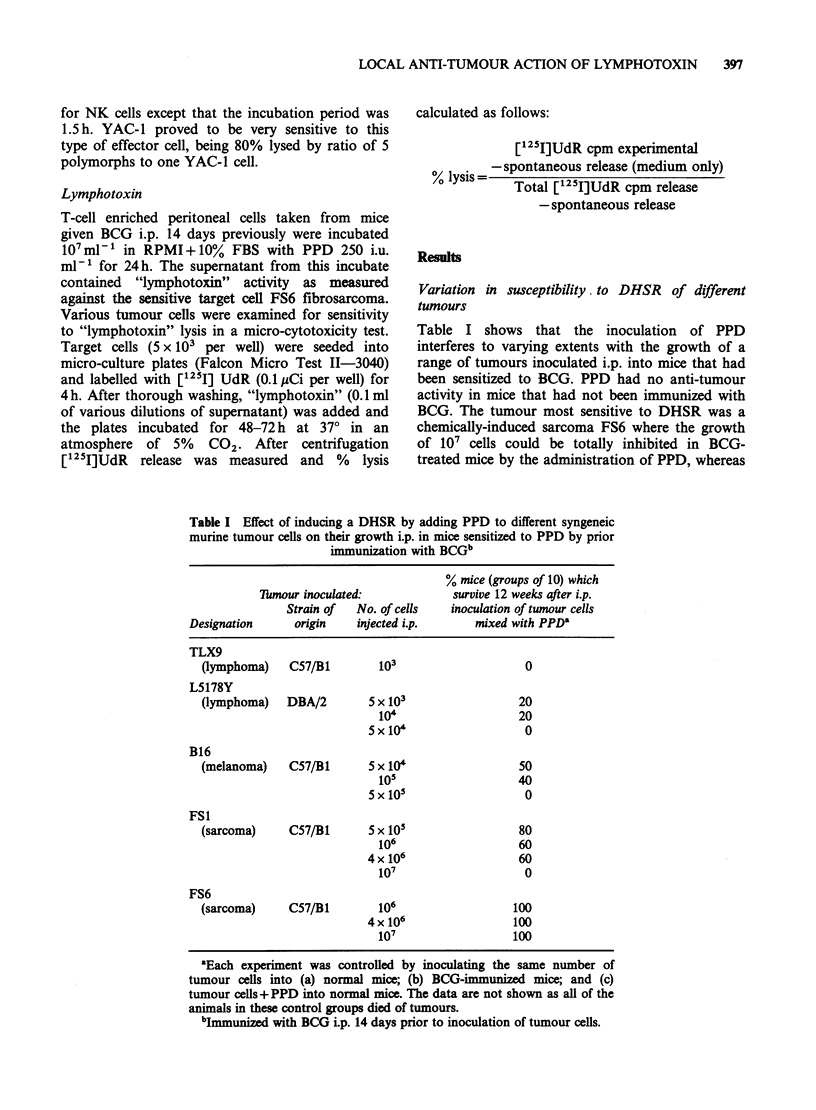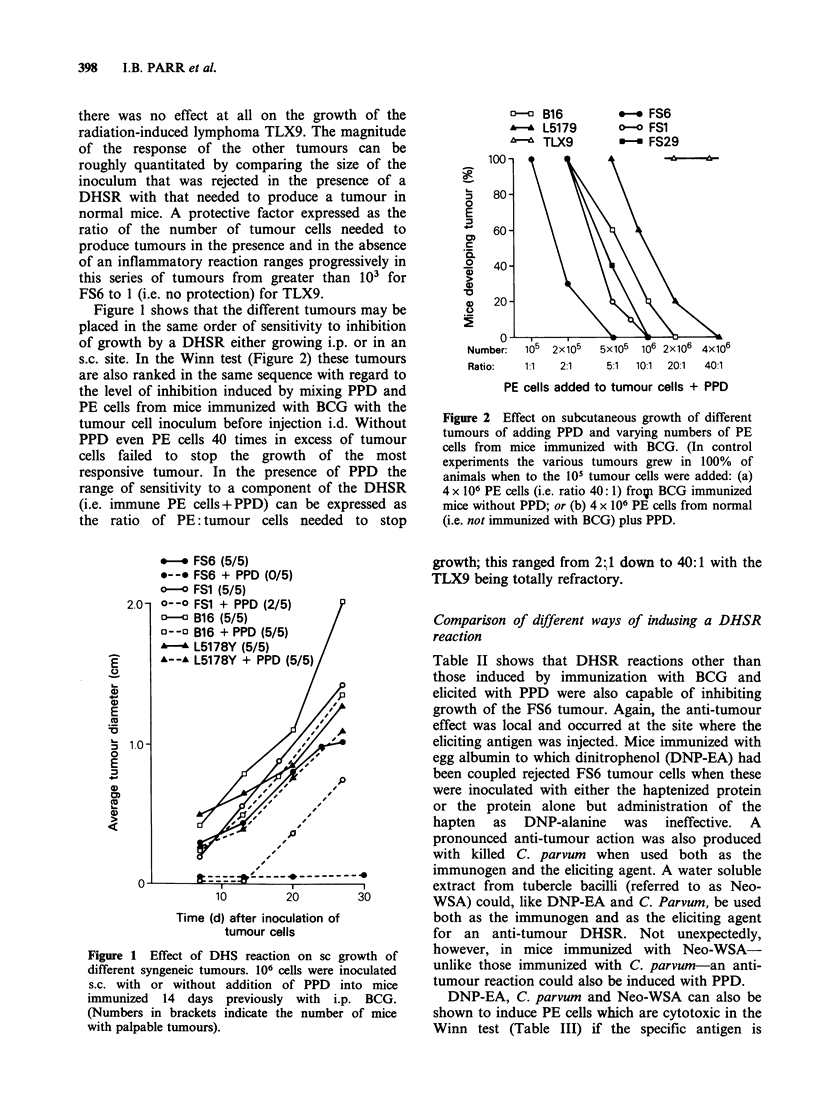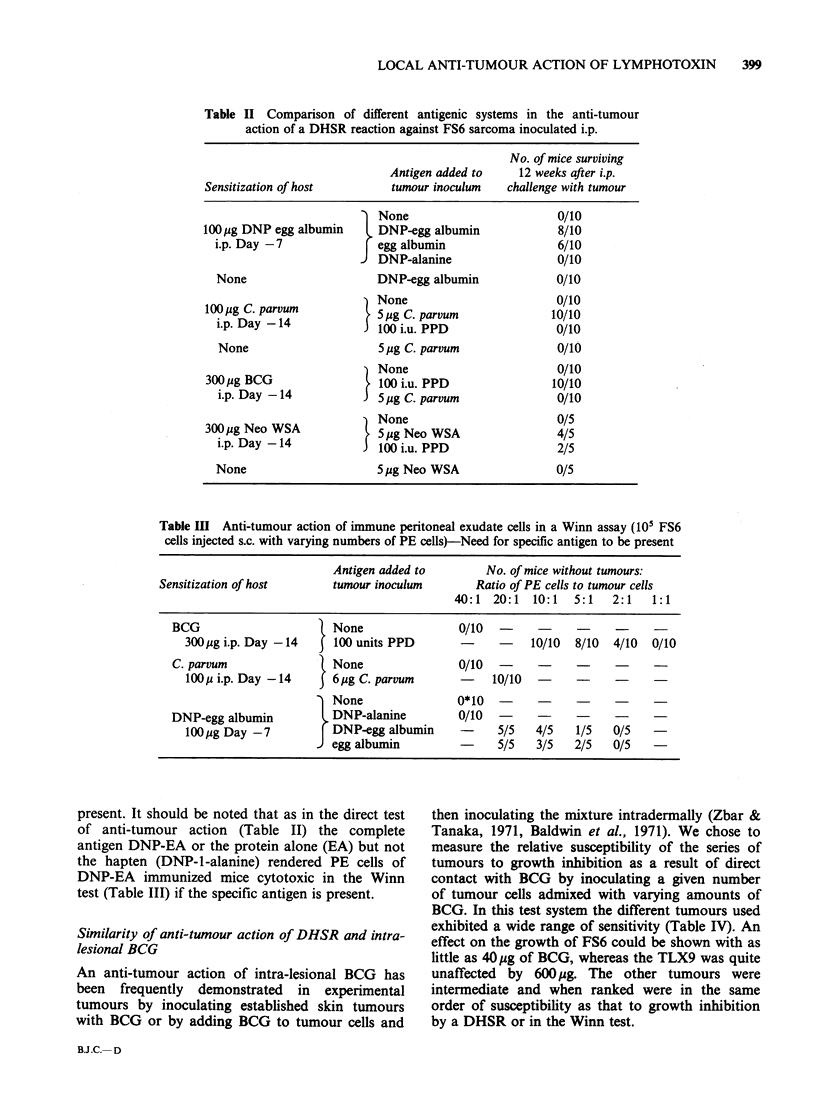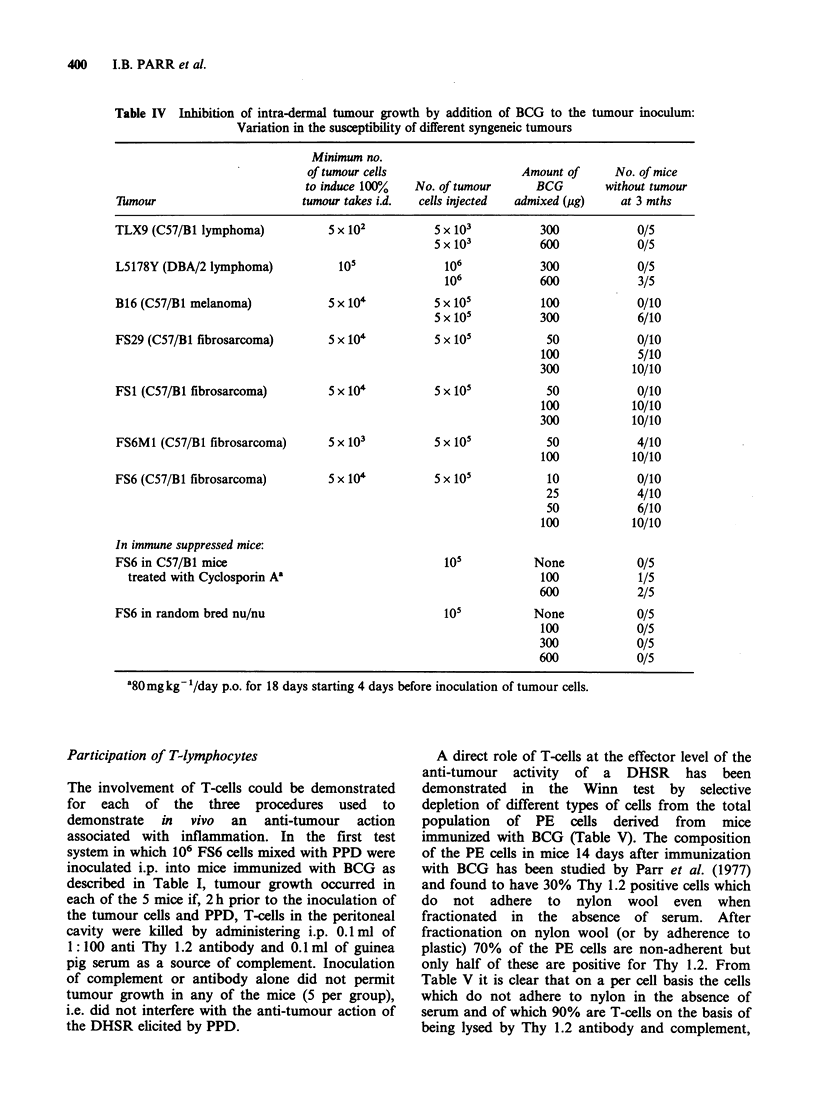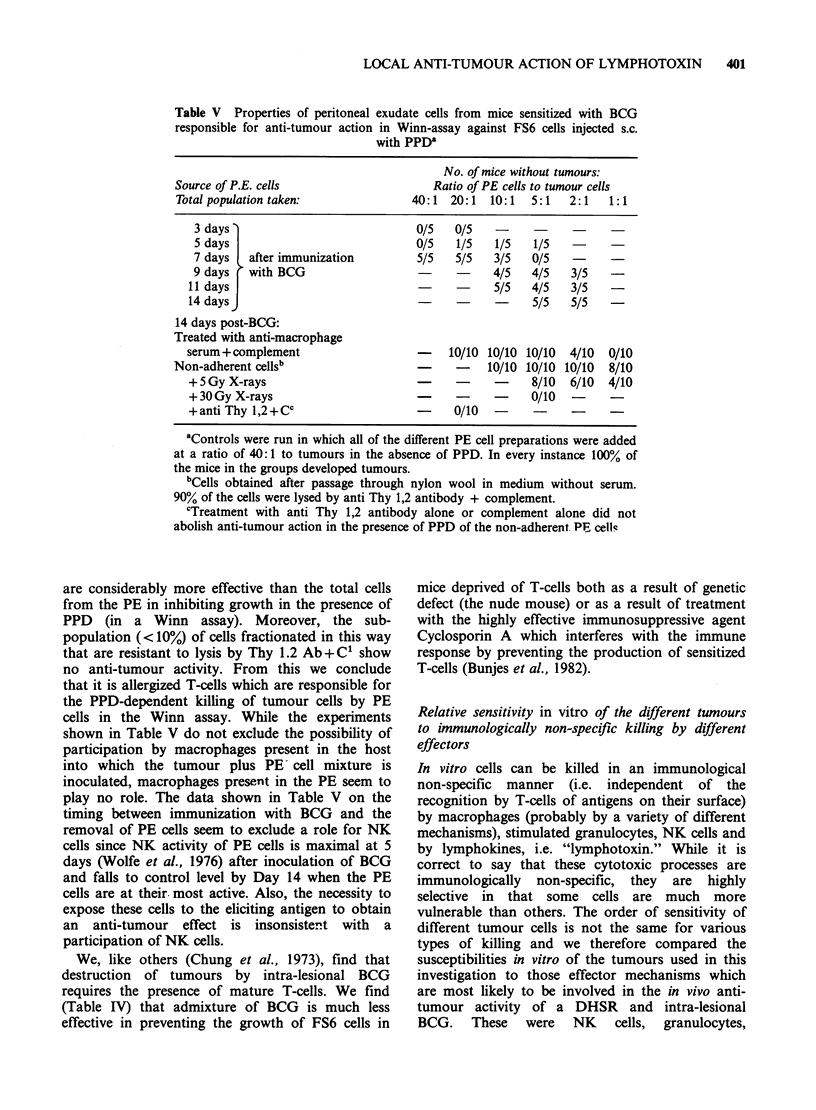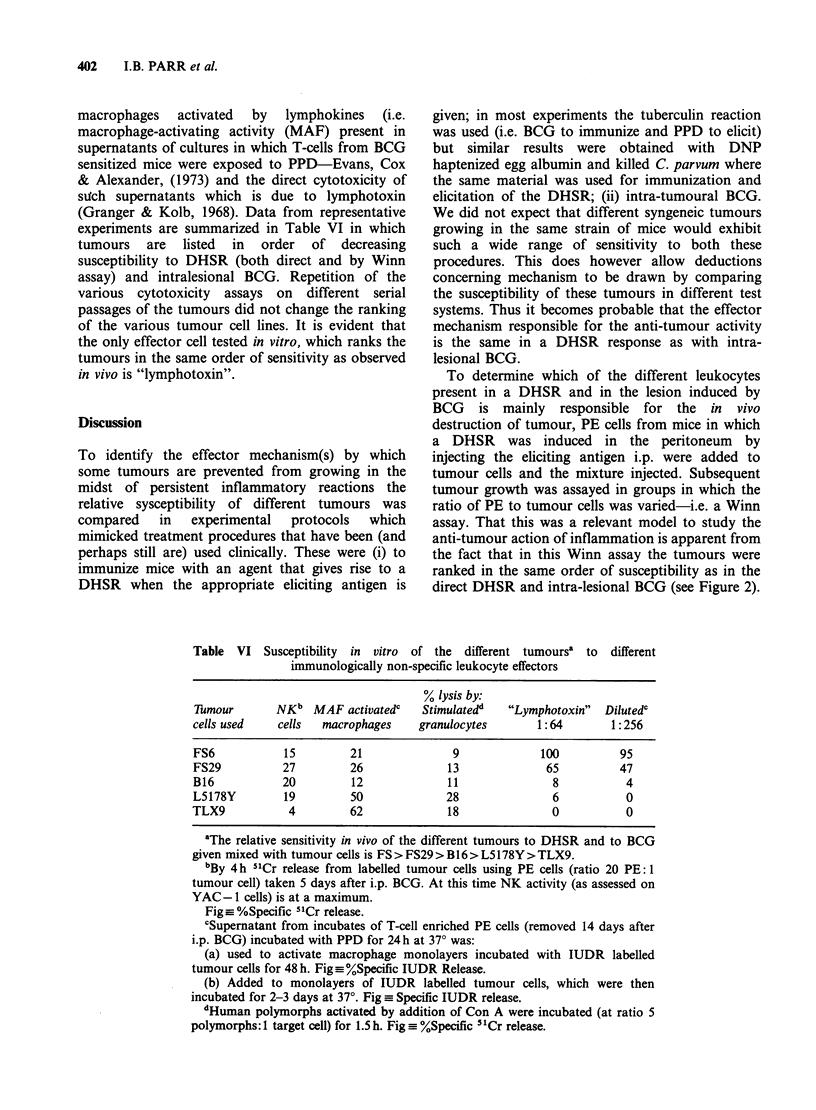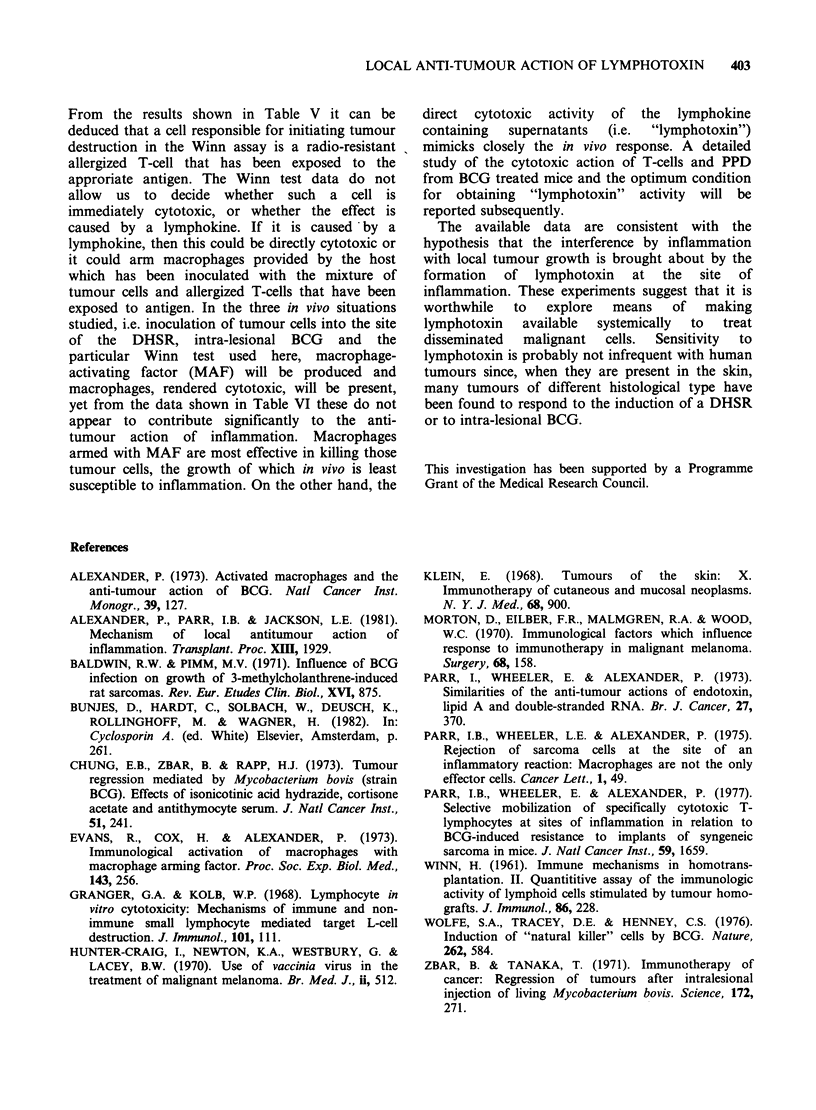# Role of "lymphotoxin" in the local anti-tumour action associated with inflammation caused by delayed hypersensitivity responses or intralesional BCG. I. Variations in response of different syngeneic mouse tumours.

**DOI:** 10.1038/bjc.1983.204

**Published:** 1983-09

**Authors:** I. B. Parr, L. E. Jackson, P. Alexander

## Abstract

The anti-tumour effect induced by a delayed hypersensitivity response (DHSR) unrelated to the tumour or by intra-tumoural inoculation of BCG was studied with 6 syngeneic mouse tumours. The growth of the tumours was followed i.p. or s.c. in suitably sensitized animals either in the presence or absence of the specific antigen required to elicit a DHSR. In a Winn-type assay the growth of tumour cells admixed with sensitized lymphocytes was also determined with and without the eliciting antigens. In addition, the effect of admixing different amounts of BCG with the tumour cells was studied on the growth of the tumours in vivo. The different tumours varied widely in their susceptibility to growth inhibition by a DHSR reaction and by BCG but their order of sensitivity was the same in all of the tests. Analysis of the effector population in the Winn test coupled with the inability to observe an anti-tumour action in mice with defective T-cell function showed that the effector mechanism involved allergized T-cells or more probably products released when these were confronted with the specific antigen. In vitro the relative susceptibility of the different tumour cells to killing by activated macrophages and by NK cells was quite different to that found for in vivo growth inhibition but the in vitro response to lymphotoxin of the different tumours paralleled that produced by inflammation in vivo.


Br. J. Cancer (1983), 48, 395–403

### Role of "lymphotoxin" in the local anti-tumour action associated with inflammation caused by delayed hypersensitivity responses or intralesional BCG I. Variations in response of different syngeneic mouse tumours

I.B. Parr, L.E. Jackson & P. Alexander[1]

*Institute of Cancer Research, Sutton, Surrey and [1]CRC Medical Oncology Unit, Southampton General Hospital, Southampton.*

**Summary** The anti-tumour effect induced by a delayed hypersensitivity response (DHSR) unrelated to the tumour or by intra-tumoural inoculation of BCG was studied with 6 syngeneic mouse tumours. The growth of the tumours was followed i.p. or s.c. in suitably sensitized animals either in the presence or absence of the specific antigen required to elicit a DHSR. In a Winn-type assay the growth of tumour cells admixed with sensitized lymphocytes was also determined with and without the eliciting antigens. In addition, the effect of admixing different amounts of BCG with the tumour cells was studied on the growth of the tumours *in vivo*. The different tumours varied widely in their susceptibility to growth inhibition by a DHSR reaction and by BCG but their order of sensitivity was the same in all of the tests.

Analysis of the effector population in the Winn test coupled with the inability to observe an anti-tumour action in mice with defective T-cell function showed that the effector mechanism involved allergized T-cells or more probably products released when these were confronted with the specific antigen. *In vitro* the relative susceptibility of the different tumour cells to killing by activated macrophages and by NK cells was quite different to that found for *in vivo* growth inhibition but the *in vitro* response to lymphotoxin of the different tumours paralleled that produced by inflammation *in vivo*.

The destruction of some local tumours—principally but not exclusively of the skin—as a result of inducing persistent inflammation by a delayed-type hypersensitivity response (DHSR) (Klein, 1968), inoculation of *vaccinia* virus (Hunter-Craig *et al.*, 1970) or intratumoural inoculation of BCG (Morton *et al.*, 1970) was first observed in man. This effect can be mimicked in rodents with some, but not all, syngeneic transplantable tumours. An *in vivo* anti-tumour action of a DHSR was demonstrated in our laboratory by immunizing mice with BCG and then injecting the eliciting antigen, purified derivative of tuberculin (PPD) either mixed with or given one or two days after, inoculation of tumour cells i.p., i.v., s.c. or i.d. (Alexander, 1973, and Alexander *et al.*, 1981).

Initially, we ascribed the destruction of tumour cells at the site of a DHSR reaction to activated macrophages (Alexander, 1973) but subsequently found (Parr *et al.*, 1975) in a Winn-type assay, in which leukocytes from an inflammatory site (peritoneal cavity) are added to tumour cells *in vitro* and the mixture inoculated into mice, that the lymphocytes and not the macrophages were the principal cytotoxic cells involved. This study raised the possibility that a cytotoxic lymphokine(s) could be the agent responsible for the destruction of tumour in the midst of an inflammatory reaction.

This investigation addressed itself to the questions: (a) is the mechanism by which a DHSR eliminates a local tumour the same as that responsible for the anti-tumour action of intra-lesional BCG; and (b) which of the potential anti-tumour agents present in a DHSR are responsible for the *in vivo* anti-tumour effect? Activated macrophages, NK cells, allergized lymphocytes or lymphokines (e.g. lymphotoxin) released when allergized lymphocytes contact the specific antigen are all likely to be present in a lesion induced by a DHSR and all have been shown to be capable of killing tumour cells *in vitro*, Alexander (1973), Wolfe *et al.* (1976), Granger & Kolb (1968).

Our approach to this problem was to compare in a series of syngeneic mouse tumours, their susceptibility to inhibition *in vivo* (i) by a DHSR (in both skin and peritoneal cavity), (ii) by intra-lesional BCG, and (iii) in a Winn assay using cells from a peritoneal exudate induced by a DHSR. The combined data strongly support the hypothesis that a cytotoxic factor elaborated when T-cells

Correspondence: P. Alexander, CRC Medical Oncology Unit, Southampton General Hospital, Southampton SO9 4XY

Received 10 February 1983; accepted 9 June 1983

© The Macmillan Press Ltd., 1983

from sensitized animals meet the specific antigen and which has the properties ascribed to lymphotoxin, is responsible for the destruction of certain tumours by both a DHSR and intra-lesional BCG.

### Materials and methods

### Animals

Ten-week old mice C57/B1 and DBA/2 were supplied by the breeding unit of the Chester Beatty Research Institute. They were fed the usual diet of water and mouse chow *ad libitum* throughout the experiments.

### Tumours

Details of fibrosarcomas FS6, FS1 (C57/B1 mice) and lymphomas L5178Y (DBA/2) and TLX9 (C57/B1) were given previously (Parr *et al.*, 1973). FS29 is a benzpyrene; induced fibrosarcoma, whilst FS6M1 is a metastatic line which arose after prolonged passage of FS6. Minimum numbers of tumour cells necessary for 100% tumour incidence (at i.d. site) are given in Table IV. All tumours were adapted to grow in either suspension (lymphomas) or monolayer (fibrosarcomas) culture and were maintained in RPMI 1640 medium supplemented with 10% FBS, sodium pyruvate and L-glutamine. Early storage of the tumour cells in liquid $N_2$ as soon as growth was established *in vitro* allowed regular returns to the tumour bank after 9–10 passages. Target cells used in cytotoxicity assays were taken from serial passage numbers 3–10.

### Chemicals

Freeze-dried BCG vaccine (percutaneous: Glaxo Laboratories Ltd., Greenford, Middlesex, England) contained $1\text{–}3\times10^8$ viable units per ampoule equivalent to 3 mg (moist weight) organisms $ml^{-1}$. The dose of BCG was 300 $\mu g$ (i.e. $\frac{1}{10}$th of an ampoule) except where stated. WSA (Water soluble fraction of mycobacterial cell walls) was a gift from Professor Ledere (Institut de Chimie des Substances Naturelle, Gif-sur-Yvette, France). Purified protein derivative (PPD) prepared from tubercle bacilli and supplied undiluted at $10^5$ units $ml^{-1}$ was obtained from Evans Medical Ltd., Speke, Liverpool. The usual dose given alone or mixed with tumour was 100 units. 2,4-dinitrophenyl-L-α-alanine was purchased from BDH Chemicals Ltd., Poole, Dorset. Egg albumin and DNP-albumin conjugate were supplied by Calbiochem-Behring Corps., P.O. Box 22, Bishops Stortford, England. *Corynebacterium parvum* was supplied by Wellcome Reagents, Beckenham, Kent as a killed saline suspension. Anti Thy 1.2-(C3H mouse monoclonal) serum was obtained from Searle Diagnostic, High Wycombe, England.

### Preparation of peritoneal exudate cells

Full details are recorded in Parr (1977). Briefly, PE cells were taken, unless stated otherwise, from mice given BCG i.p. 14 days previously, using cooled siliconized glassware. Nylon-wool fractionated cells prepared in the absence of serum yielded a population of small lymphocytes, 90% of which were killed by incubation with anti Thy 1.2 and guinea pig complement. On the other hand cell preparations containing NK cell activity came from mice given BCG 3 days before. These peritoneal cells were fractionated on nylon-wool columns in the presence of serum.

### Winn assay

As described, Winn (1961) and Parr *et al.*, (1977) tumour cells were added *in vitro* to effector cells at various ratios and the cell mixture injected s.c. into syngeneic mice.

### NK cell assay

Target cells at $10^7\,ml^{-1}$ were labelled with $Na_2{}^{51}CrO_4$ (100 $\mu Ci$) for 1 h at 37°, after which time the cells were washed ×3 with medium 199, resuspended in RPMI-1640 and incubated for a further hour at 37°. After washing, the cells $10^4$/well were placed in Nunc-u-bottomed microtest plates with varying numbers of peritoneal exudate cells (non-adherent fraction) and incubated for 4 h at 37° in a 5% $CO_2$ atmosphere. $^{51}Cr$ was determined in 0.1 ml of supernate (plate was centrifuged at 200 g for 5 min) using a Packacd auto-gamma counter. Per cent specific lysis was calculated as follows:

$$=\frac{{}^{51}\text{Cr cpm experimental} - \text{spontaneous release (medium only)}}{\text{Maximal release (freezing and thawing)} - \text{spontaneous release}}\times 100$$

The NK sensitive target-cell line YAC-1 incubated under the above conditions with PE cells at an effector: target ratio of 20:1 gave 50% specific lysis.

### Stimulated granulocytes

Human polymorphonuclear leucotes prepared from whole blood by sedimentation through dextran (5%) were activated by addition of Concanavalin A(5 $\mu g$ per well). Assay conditions were the same as for NK cells except that the incubation period was 1.5 h. YAC-1 proved to be very sensitive to this type of effector cell, being 80% lysed by ratio of 5 polymorphs to one YAC-1 cell.

*Lymphotoxin*

T-cell enriched peritoneal cells taken from mice given BCG i.p. 14 days previously were incubated $10^7 ml^{-1}$ in RPMI+10% FBS with PPD 250 i.u. $ml^{-1}$ for 24 h. The supernatant from this incubate contained "lymphotoxin" activity as measured against the sensitive target cell FS6 fibrosarcoma. Various tumour cells were examined for sensitivity to "lymphotoxin" lysis in a micro-cytotoxicity test. Target cells ($5 \times 10^3$ per well) were seeded into micro-culture plates (Falcon Micro Test II—3040) and labelled with $[^{125}I]$ UdR (0.1 μCi per well) for 4 h. After thorough washing, "lymphotoxin" (0.1 ml of various dilutions of supernatant) was added and the plates incubated for 48–72 h at 37° in an atmosphere of 5% $CO_2$. After centrifugation $[^{125}I]$UdR release was measured and % lysis calculated as follows:

$$\% \text{ lysis} = \frac{[^{125}I]\text{UdR cpm experimental} - \text{spontaneous release (medium only)}}{\text{Total } [^{125}I]\text{UdR cpm release} - \text{spontaneous release}}$$

### Results

*Variation in susceptibility to DHSR of different tumours*

Table I shows that the inoculation of PPD interferes to varying extents with the growth of a range of tumours inoculated i.p. into mice that had been sensitized to BCG. PPD had no anti-tumour activity in mice that had not been immunized with BCG. The tumour most sensitive to DHSR was a chemically-induced sarcoma FS6 where the growth of $10^7$ cells could be totally inhibited in BCG-treated mice by the administration of PPD, whereas

**Table I** Effect of inducing a DHSR by adding PPD to different syngeneic murine tumour cells on their growth i.p. in mice sensitized to PPD by prior immunization with BCG[b]

| *Tumour inoculated:* *Designation* | *Strain of origin* | *No. of cells injected i.p.* | *% mice (groups of 10) which survive 12 weeks after i.p. inoculation of tumour cells mixed with PPD*[a] |
|---|---|---|---|
| TLX9 (lymphoma) | C57/B1 | $10^3$ | 0 |
| L5178Y (lymphoma) | DBA/2 | $5 \times 10^3$ | 20 |
| | | $10^4$ | 20 |
| | | $5 \times 10^4$ | 0 |
| B16 (melanoma) | C57/B1 | $5 \times 10^4$ | 50 |
| | | $10^5$ | 40 |
| | | $5 \times 10^5$ | 0 |
| FS1 (sarcoma) | C57/B1 | $5 \times 10^5$ | 80 |
| | | $10^6$ | 60 |
| | | $4 \times 10^6$ | 60 |
| | | $10^7$ | 0 |
| FS6 (sarcoma) | C57/B1 | $10^6$ | 100 |
| | | $4 \times 10^6$ | 100 |
| | | $10^7$ | 100 |

[a]Each experiment was controlled by inoculating the same number of tumour cells into (a) normal mice; (b) BCG-immunized mice; and (c) tumour cells+PPD into normal mice. The data are not shown as all of the animals in these control groups died of tumours.

[b]Immunized with BCG i.p. 14 days prior to inoculation of tumour cells.

there was no effect at all on the growth of the radiation-induced lymphoma TLX9. The magnitude of the response of the other tumours can be roughly quantitated by comparing the size of the inoculum that was rejected in the presence of a DHSR with that needed to produce a tumour in normal mice. A protective factor expressed as the ratio of the number of tumour cells needed to produce tumours in the presence and in the absence of an inflammatory reaction ranges progressively in this series of tumours from greater than $10^3$ for FS6 to 1 (i.e. no protection) for TLX9.

Figure 1 shows that the different tumours may be placed in the same order of sensitivity to inhibition of growth by a DHSR either growing i.p. or in an s.c. site. In the Winn test (Figure 2) these tumours are also ranked in the same sequence with regard to the level of inhibition induced by mixing PPD and PE cells from mice immunized with BCG with the tumour cell inoculum before injection i.d. Without PPD even PE cells 40 times in excess of tumour cells failed to stop the growth of the most responsive tumour. In the presence of PPD the range of sensitivity to a component of the DHSR (i.e. immune PE cells + PPD) can be expressed as the ratio of PE : tumour cells needed to stop growth; this ranged from 2:1 down to 40:1 with the TLX9 being totally refractory.

**Figure 2** Effect on subcutaneous growth of different tumours of adding PPD and varying numbers of PE cells from mice immunized with BCG. (In control experiments the various tumours grew in 100% of animals when to the $10^5$ tumour cells were added: (a) $4\times10^6$ PE cells (i.e. ratio 40 : 1) from BCG immunized mice without PPD; *or* (b) $4\times10^6$ PE cells from normal (i.e. *not* immunized with BCG) plus PPD.

**Figure 1** Effect of DHS reaction on sc growth of different syngeneic tumours. $10^6$ cells were inoculated s.c. with or without addition of PPD into mice immunized 14 days previously with i.p. BCG. (Numbers in brackets indicate the number of mice with palpable tumours).

### *Comparison of different ways of indusing a DHSR reaction*

Table II shows that DHSR reactions other than those induced by immunization with BCG and elicited with PPD were also capable of inhibiting growth of the FS6 tumour. Again, the anti-tumour effect was local and occurred at the site where the eliciting antigen was injected. Mice immunized with egg albumin to which dinitrophenol (DNP-EA) had been coupled rejected FS6 tumour cells when these were inoculated with either the haptenized protein or the protein alone but administration of the hapten as DNP-alanine was ineffective. A pronounced anti-tumour action was also produced with killed *C. parvum* when used both as the immunogen and the eliciting agent. A water soluble extract from tubercle bacilli (referred to as Neo-WSA) could, like DNP-EA and *C. Parvum*, be used both as the immunogen and as the eliciting agent for an anti-tumour DHSR. Not unexpectedly, however, in mice immunized with Neo-WSA—unlike those immunized with *C. parvum*—an anti-tumour reaction could also be induced with PPD.

DNP-EA, *C. parvum* and Neo-WSA can also be shown to induce PE cells which are cytotoxic in the Winn test (Table III) if the specific antigen is

**Table II** Comparison of different antigenic systems in the anti-tumour action of a DHSR reaction against FS6 sarcoma inoculated i.p.

| *Sensitization of host* | *Antigen added to tumour inoculum* | *No. of mice surviving 12 weeks after i.p. challenge with tumour* |
|---|---|---|
| 100 μg DNP egg albumin i.p. Day −7 | None | 0/10 |
| | DNP-egg albumin | 8/10 |
| | egg albumin | 6/10 |
| | DNP-alanine | 0/10 |
| None | DNP-egg albumin | 0/10 |
| 100 μg *C. parvum* i.p. Day −14 | None | 0/10 |
| | 5 μg *C. parvum* | 10/10 |
| | 100 i.u. PPD | 0/10 |
| None | 5 μg *C. parvum* | 0/10 |
| 300 μg BCG i.p. Day −14 | None | 0/10 |
| | 100 i.u. PPD | 10/10 |
| | 5 μg *C. parvum* | 0/10 |
| 300 μg Neo WSA i.p. Day −14 | None | 0/5 |
| | 5 μg Neo WSA | 4/5 |
| | 100 i.u. PPD | 2/5 |
| None | 5 μg Neo WSA | 0/5 |

**Table III** Anti-tumour action of immune peritoneal exudate cells in a Winn assay ($10^5$ FS6 cells injected s.c. with varying numbers of PE cells)—Need for specific antigen to be present

| *Sensitization of host* | *Antigen added to tumour inoculum* | *No. of mice without tumours: Ratio of PE cells to tumour cells* | | | | | |
|---|---|---|---|---|---|---|---|
| | | 40:1 | 20:1 | 10:1 | 5:1 | 2:1 | 1:1 |
| BCG 300 μg i.p. Day −14 | None | 0/10 | — | — | — | — | — |
| | 100 units PPD | — | — | 10/10 | 8/10 | 4/10 | 0/10 |
| *C. parvum* 100 μ i.p. Day −14 | None | 0/10 | — | — | — | — | — |
| | 6 μg *C. parvum* | — | 10/10 | — | — | — | — |
| DNP-egg albumin 100 μg Day −7 | None | 0*10 | — | — | — | — | — |
| | DNP-alanine | 0/10 | — | — | — | — | — |
| | DNP-egg albumin | — | 5/5 | 4/5 | 1/5 | 0/5 | — |
| | egg albumin | — | 5/5 | 3/5 | 2/5 | 0/5 | — |

present. It should be noted that as in the direct test of anti-tumour action (Table II) the complete antigen DNP-EA or the protein alone (EA) but not the hapten (DNP-1-alanine) rendered PE cells of DNP-EA immunized mice cytotoxic in the Winn test (Table III) if the specific antigen is present.

### *Similarity of anti-tumour action of DHSR and intra-lesional BCG*

An anti-tumour action of intra-lesional BCG has been frequently demonstrated in experimental tumours by inoculating established skin tumours with BCG or by adding BCG to tumour cells and then inoculating the mixture intradermally (Zbar & Tanaka, 1971, Baldwin *et al.*, 1971). We chose to measure the relative susceptibility of the series of tumours to growth inhibition as a result of direct contact with BCG by inoculating a given number of tumour cells admixed with varying amounts of BCG. In this test system the different tumours used exhibited a wide range of sensitivity (Table IV). An effect on the growth of FS6 could be shown with as little as 40 μg of BCG, whereas the TLX9 was quite unaffected by 600 μg. The other tumours were intermediate and when ranked were in the same order of susceptibility as that to growth inhibition by a DHSR or in the Winn test.

**Table IV** Inhibition of intra-dermal tumour growth by addition of BCG to the tumour inoculum: Variation in the susceptibility of different syngeneic tumours

| *Tumour* | *Minimum no. of tumour cells to induce 100% tumour takes i.d.* | *No. of tumour cells injected* | *Amount of BCG admixed (μg)* | *No. of mice without tumour at 3 mths* |
|---|---|---|---|---|
| TLX9 (C57/B1 lymphoma) | $5 \times 10^2$ | $5 \times 10^3$ | 300 | 0/5 |
| | | $5 \times 10^3$ | 600 | 0/5 |
| L5178Y (DBA/2 lymphoma) | $10^5$ | $10^6$ | 300 | 0/5 |
| | | $10^6$ | 600 | 3/5 |
| B16 (C57/B1 melanoma) | $5 \times 10^4$ | $5 \times 10^5$ | 100 | 0/10 |
| | | $5 \times 10^5$ | 300 | 6/10 |
| FS29 (C57/B1 fibrosarcoma) | $5 \times 10^4$ | $5 \times 10^5$ | 50 | 0/10 |
| | | | 100 | 5/10 |
| | | | 300 | 10/10 |
| FS1 (C57/B1 fibrosarcoma) | $5 \times 10^4$ | $5 \times 10^5$ | 50 | 0/10 |
| | | | 100 | 10/10 |
| | | | 300 | 10/10 |
| FS6M1 (C57/B1 fibrosarcoma) | $5 \times 10^3$ | $5 \times 10^5$ | 50 | 4/10 |
| | | | 100 | 10/10 |
| FS6 (C57/B1 fibrosarcoma) | $5 \times 10^4$ | $5 \times 10^5$ | 10 | 0/10 |
| | | | 25 | 4/10 |
| | | | 50 | 6/10 |
| | | | 100 | 10/10 |
| *In immune suppressed mice*: | | | | |
| FS6 in C57/B1 mice treated with Cyclosporin A[a] | | $10^5$ | None | 0/5 |
| | | | 100 | 1/5 |
| | | | 600 | 2/5 |
| FS6 in random bred nu/nu | | $10^5$ | None | 0/5 |
| | | | 100 | 0/5 |
| | | | 300 | 0/5 |
| | | | 600 | 0/5 |

[a] $80\,\mathrm{mg\,kg^{-1}}$/day p.o. for 18 days starting 4 days before inoculation of tumour cells.

*Participation of T-lymphocytes*

The involvement of T-cells could be demonstrated for each of the three procedures used to demonstrate *in vivo* an anti-tumour action associated with inflammation. In the first test system in which $10^6$ FS6 cells mixed with PPD were inoculated i.p. into mice immunized with BCG as described in Table I, tumour growth occurred in each of the 5 mice if, 2 h prior to the inoculation of the tumour cells and PPD, T-cells in the peritoneal cavity were killed by administering i.p. 0.1 ml of 1:100 anti Thy 1.2 antibody and 0.1 ml of guinea pig serum as a source of complement. Inoculation of complement or antibody alone did not permit tumour growth in any of the mice (5 per group), i.e. did not interfere with the anti-tumour action of the DHSR elicited by PPD.

A direct role of T-cells at the effector level of the anti-tumour activity of a DHSR has been demonstrated in the Winn test by selective depletion of different types of cells from the total population of PE cells derived from mice immunized with BCG (Table V). The composition of the PE cells in mice 14 days after immunization with BCG has been studied by Parr *et al.* (1977) and found to have 30% Thy 1.2 positive cells which do not adhere to nylon wool even when fractionated in the absence of serum. After fractionation on nylon wool (or by adherence to plastic) 70% of the PE cells are non-adherent but only half of these are positive for Thy 1.2. From Table V it is clear that on a per cell basis the cells which do not adhere to nylon in the absence of serum and of which 90% are T-cells on the basis of being lysed by Thy 1.2 antibody and complement,

**Table V** Properties of peritoneal exudate cells from mice sensitized with BCG responsible for anti-tumour action in Winn-assay against FS6 cells injected s.c. with PPD[a]

| *Source of P.E. cells* *Total population taken:* | *No. of mice without tumours: Ratio of PE cells to tumour cells* 40:1 | 20:1 | 10:1 | 5:1 | 2:1 | 1:1 |
|---|---|---|---|---|---|---|
| 3 days after immunization with BCG | 0/5 | 0/5 | — | — | — | — |
| 5 days after immunization with BCG | 0/5 | 1/5 | 1/5 | 1/5 | — | — |
| 7 days after immunization with BCG | 5/5 | 5/5 | 3/5 | 0/5 | — | — |
| 9 days after immunization with BCG | — | — | 4/5 | 4/5 | 3/5 | — |
| 11 days after immunization with BCG | — | — | 5/5 | 4/5 | 3/5 | — |
| 14 days after immunization with BCG | — | — | — | 5/5 | 5/5 | — |
| 14 days post-BCG: | | | | | | |
| Treated with anti-macrophage serum + complement | — | 10/10 | 10/10 | 10/10 | 4/10 | 0/10 |
| Non-adherent cells[b] | — | — | 10/10 | 10/10 | 10/10 | 8/10 |
| +5 Gy X-rays | — | — | — | 8/10 | 6/10 | 4/10 |
| +30 Gy X-rays | — | — | — | 0/10 | — | — |
| +anti Thy 1,2+C[c] | — | 0/10 | — | — | — | — |

[a]Controls were run in which all of the different PE cell preparations were added at a ratio of 40:1 to tumours in the absence of PPD. In every instance 100% of the mice in the groups developed tumours.

[b]Cells obtained after passage through nylon wool in medium without serum. 90% of the cells were lysed by anti Thy 1,2 antibody + complement.

[c]Treatment with anti Thy 1,2 antibody alone or complement alone did not abolish anti-tumour action in the presence of PPD of the non-adherent PE cells are considerably more effective than the total cells from the PE in inhibiting growth in the presence of PPD (in a Winn assay). Moreover, the sub-population (<10%) of cells fractionated in this way that are resistant to lysis by Thy 1.2 Ab+C[1] show no anti-tumour activity. From this we conclude that it is allergized T-cells which are responsible for the PPD-dependent killing of tumour cells by PE cells in the Winn assay. While the experiments shown in Table V do not exclude the possibility of participation by macrophages present in the host into which the tumour plus PE cell mixture is inoculated, macrophages present in the PE seem to play no role. The data shown in Table V on the timing between immunization with BCG and the removal of PE cells seem to exclude a role for NK cells since NK activity of PE cells is maximal at 5 days (Wolfe *et al.*, 1976) after inoculation of BCG and falls to control level by Day 14 when the PE cells are at their most active. Also, the necessity to expose these cells to the eliciting antigen to obtain an anti-tumour effect is insonsistent with a participation of NK cells.

We, like others (Chung *et al.*, 1973), find that destruction of tumours by intra-lesional BCG requires the presence of mature T-cells. We find (Table IV) that admixture of BCG is much less effective in preventing the growth of FS6 cells in mice deprived of T-cells both as a result of genetic defect (the nude mouse) or as a result of treatment with the highly effective immunosuppressive agent Cyclosporin A which interferes with the immune response by preventing the production of sensitized T-cells (Bunjes *et al.*, 1982).

*Relative sensitivity* in vitro *of the different tumours to immunologically non-specific killing by different effectors*

*In vitro* cells can be killed in an immunological non-specific manner (i.e. independent of the recognition by T-cells of antigens on their surface) by macrophages (probably by a variety of different mechanisms), stimulated granulocytes, NK cells and by lymphokines, i.e. "lymphotoxin." While it is correct to say that these cytotoxic processes are immunologically non-specific, they are highly selective in that some cells are much more vulnerable than others. The order of sensitivity of different tumour cells is not the same for various types of killing and we therefore compared the susceptibilities *in vitro* of the tumours used in this investigation to those effector mechanisms which are most likely to be involved in the *in vivo* anti-tumour activity of a DHSR and intra-lesional BCG. These were NK cells, granulocytes, macrophages activated by lymphokines (i.e. macrophage-activating activity (MAF) present in supernatants of cultures in which T-cells from BCG sensitized mice were exposed to PPD—Evans, Cox & Alexander, (1973) and the direct cytotoxicity of such supernatants which is due to lymphotoxin (Granger & Kolb, 1968). Data from representative experiments are summarized in Table VI in which tumours are listed in order of decreasing susceptibility to DHSR (both direct and by Winn assay) and intralesional BCG. Repetition of the various cytotoxicity assays on different serial passages of the tumours did not change the ranking of the various tumour cell lines. It is evident that the only effector cell tested *in vitro,* which ranks the tumours in the same order of sensitivity as observed *in vivo* is "lymphotoxin".

### Discussion

To identify the effector mechanism(s) by which some tumours are prevented from growing in the midst of persistent inflammatory reactions the relative sysceptibility of different tumours was compared in experimental protocols which mimicked treatment procedures that have been (and perhaps still are) used clinically. These were (i) to immunize mice with an agent that gives rise to a DHSR when the appropriate eliciting antigen is given; in most experiments the tuberculin reaction was used (i.e. BCG to immunize and PPD to elicit) but similar results were obtained with DNP haptenized egg albumin and killed *C. parvum* where the same material was used for immunization and elicitation of the DHSR; (ii) intra-tumoural BCG. We did not expect that different syngeneic tumours growing in the same strain of mice would exhibit such a wide range of sensitivity to both these procedures. This does however allow deductions concerning mechanism to be drawn by comparing the susceptibility of these tumours in different test systems. Thus it becomes probable that the effector mechanism responsible for the anti-tumour activity is the same in a DHSR response as with intra-lesional BCG.

To determine which of the different leukocytes present in a DHSR and in the lesion induced by BCG is mainly responsible for the *in vivo* destruction of tumour, PE cells from mice in which a DHSR was induced in the peritoneum by injecting the eliciting antigen i.p. were added to tumour cells and the mixture injected. Subsequent tumour growth was assayed in groups in which the ratio of PE to tumour cells was varied—i.e. a Winn assay. That this was a relevant model to study the anti-tumour action of inflammation is apparent from the fact that in this Winn assay the tumours were ranked in the same order of susceptibility as in the direct DHSR and intra-lesional BCG (see Figure 2).

**Table VI** Susceptibility *in vitro* of the different tumours[a] to different immunologically non-specific leukocyte effectors

| | | | % lysis by: | | |
|---|---|---|---|---|---|
| *Tumour cells used* | *NK*[b] *cells* | *MAF activated*[c] *macrophages* | *Stimulated*[d] *granulocytes* | *"Lymphotoxin"* 1:64 | *Diluted*[c] 1:256 |
| FS6 | 15 | 21 | 9 | 100 | 95 |
| FS29 | 27 | 26 | 13 | 65 | 47 |
| B16 | 20 | 12 | 11 | 8 | 4 |
| L5178Y | 19 | 50 | 28 | 6 | 0 |
| TLX9 | 4 | 62 | 18 | 0 | 0 |

[a]The relative sensitivity *in vivo* of the different tumours to DHSR and to BCG given mixed with tumour cells is FS > FS29 > B16 > L5178Y > TLX9.

[b]By 4 h $^{51}$Cr release from labelled tumour cells using PE cells (ratio 20 PE:1 tumour cell) taken 5 days after i.p. BCG. At this time NK activity (as assessed on YAC−1 cells) is at a maximum.

Fig ≡ %Specific $^{51}$Cr release.

[c]Supernatant from incubates of T-cell enriched PE cells (removed 14 days after i.p. BCG) incubated with PPD for 24 h at 37° was:

(a) used to activate macrophage monolayers incubated with IUDR labelled tumour cells for 48 h. Fig ≡ %Specific IUDR Release.

(b) Added to monolayers of IUDR labelled tumour cells, which were then incubated for 2–3 days at 37°. Fig ≡ Specific IUDR release.

[d]Human polymorphs activated by addition of Con A were incubated (at ratio 5 polymorphs:1 target cell) for 1.5 h. Fig ≡ %Specific $^{51}$Cr release.

From the results shown in Table V it can be deduced that a cell responsible for initiating tumour destruction in the Winn assay is a radio-resistant allergized T-cell that has been exposed to the approriate antigen. The Winn test data do not allow us to decide whether such a cell is immediately cytotoxic, or whether the effect is caused by a lymphokine. If it is caused by a lymphokine, then this could be directly cytotoxic or it could arm macrophages provided by the host which has been inoculated with the mixture of tumour cells and allergized T-cells that have been exposed to antigen. In the three *in vivo* situations studied, i.e. inoculation of tumour cells into the site of the DHSR, intra-lesional BCG and the particular Winn test used here, macrophage-activating factor (MAF) will be produced and macrophages, rendered cytotoxic, will be present, yet from the data shown in Table VI these do not appear to contribute significantly to the anti-tumour action of inflammation. Macrophages armed with MAF are most effective in killing those tumour cells, the growth of which *in vivo* is least susceptible to inflammation. On the other hand, the direct cytotoxic activity of the lymphokine containing supernatants (i.e. "lymphotoxin") mimicks closely the *in vivo* response. A detailed study of the cytotoxic action of T-cells and PPD from BCG treated mice and the optimum condition for obtaining "lymphotoxin" activity will be reported subsequently.

The available data are consistent with the hypothesis that the interference by inflammation with local tumour growth is brought about by the formation of lymphotoxin at the site of inflammation. These experiments suggest that it is worthwhile to explore means of making lymphotoxin available systemically to treat disseminated malignant cells. Sensitivity to lymphotoxin is probably not infrequent with human tumours since, when they are present in the skin, many tumours of different histological type have been found to respond to the induction of a DHSR or to intra-lesional BCG.

This investigation has been supported by a Programme Grant of the Medical Research Council.

### References

ALEXANDER, P. (1973). Activated macrophages and the anti-tumour action of BCG. *Natl Cancer Inst. Monogr.*, **39**, 127.

ALEXANDER, P., PARR, I.B. & JACKSON, L.E. (1981). Mechanism of local antitumour action of inflammation. *Transplant. Proc.* **XIII**, 1929.

BALDWIN, R.W. & PIMM, M.V. (1971). Influence of BCG infection on growth of 3-methylcholanthrene-induced rat sarcomas. *Rev. Eur. Etudes Clin. Biol.*, **XVI**, 875.

BUNJES, D., HARDT, C., SOLBACH, W., DEUSCH, K., ROLLINGHOFF, M. & WAGNER, H. (1982). In: *Cyclosporin A.* (ed. White) Elsevier, Amsterdam, p. 261.

CHUNG, E.B., ZBAR, B. & RAPP, H.J. (1973). Tumour regression mediated by *Mycobacterium bovis* (strain BCG). Effects of isonicotinic acid hydrazide, cortisone acetate and antithymocyte serum. *J. Natl Cancer Inst.*, **51**, 241.

EVANS, R., COX, H. & ALEXANDER, P. (1973). Immunological activation of macrophages with macrophage arming factor. *Proc. Soc. Exp. Biol. Med.*, **143**, 256.

GRANGER, G.A. & KOLB, W.P. (1968). Lymphocyte *in vitro* cytotoxicity: Mechanisms of immune and non-immune small lymphocyte mediated target L-cell destruction. *J. Immunol.*, **101**, 111.

HUNTER-CRAIG, I., NEWTON, K.A., WESTBURY, G. & LACEY, B.W. (1970). Use of *vaccinia* virus in the treatment of malignant melanoma. *Br. Med. J.*, **ii**, 512.

KLEIN, E. (1968). Tumours of the skin: X. Immunotherapy of cutaneous and mucosal neoplasms. *N. Y. J. Med.*, **68**, 900.

MORTON, D., EILBER, F.R., MALMGREN, R.A. & WOOD, W.C. (1970). Immunological factors which influence response to immunotherapy in malignant melanoma. *Surgery*, **68**, 158.

PARR, I., WHEELER, E. & ALEXANDER, P. (1973). Similarities of the anti-tumour actions of endotoxin, lipid A and double-stranded RNA. *Br. J. Cancer*, **27**, 370.

PARR, I.B., WHEELER, L.E. & ALEXANDER, P. (1975). Rejection of sarcoma cells at the site of an inflammatory reaction: Macrophages are not the only effector cells. *Cancer Lett.*, **1**, 49.

PARR, I.B., WHEELER, E. & ALEXANDER, P. (1977). Selective mobilization of specifically cytotoxic T-lymphocytes at sites of inflammation in relation to BCG-induced resistance to implants of syngeneic sarcoma in mice. *J. Natl Cancer Inst.*, **59**, 1659.

WINN, H. (1961). Immune mechanisms in homotransplantation. II. Quantititive assay of the immunologic activity of lymphoid cells stimulated by tumour homografts. *J. Immunol.*, **86**, 228.

WOLFE, S.A., TRACEY, D.E. & HENNEY, C.S. (1976). Induction of "natural killer" cells by BCG. *Nature*, **262**, 584.

ZBAR, B. & TANAKA, T. (1971). Immunotherapy of cancer: Regression of tumours after intralesional injection of living *Mycobacterium bovis*. *Science*, **172**, 271.